# Using a SCIP-PLUS perspective to reduce the risk of SSI

**DOI:** 10.1186/1753-6561-5-S6-O55

**Published:** 2011-06-29

**Authors:** CE Edmiston, CJ Krepel, GR Seabrook

**Affiliations:** 1 Surgery, Medical College of Wisconsin, Milwaukee, Wisconsin, USA

## Introduction / objectives

Recent findings suggest that the Surgical Care Improvement Project (SCIP) a US-Based “process initiative” fails to reduce the risk of SSIs. The following evidence-based discussion proposes a SCIP-PLUS perspective for reducing risk of SSIs through enhanced antimicrobial prophylaxis and embracing innovative antimicrobial risk reduction technology.

## Methods

Four risk reduction initiatives were studied, A: Impact of BMI on antimicrobial prophylactic dosing in general, OB and CT surgical patients; B: Development of standardized regimen of CHG preadmission cleansing to improve outcome in surgical patients; C: Efficacy of antimicrobial sutures to reduce the risk of suture contamination at wound closure and D: The impact of an innovative antimicrobial surgical glove to reduce the risk of microbial contamination following glove microperforation.

## Results

A: 2-gm dosing in surgical patients failed to provide adequate tissue concentrations at BMI >30 (*p*<*0.05*); B: Standardization of skin cleansing using a 2% CHG polyester cloths was effective at providing skin concentrations sufficient to inhibit/kill wound pathogens compared to non-standardized regimen (*p*<*0.001*); C: Laboratory/clinical studies demonstrate that antimicrobial suture technology is effective (*p*<*0.05*) at reducing the risk of suture contamination and SSI; D: Innovative antimicrobial surgical glove was effective (*p*<*0.001*) at reducing bacterial passage following microperforation which can lead to wound contamination.

## Conclusion

An effective SCIP-PLUS strategy requires multi-faceted evidence-based approach including antimicrobial dosing to compensate for BMI, thoughtful preadmission skin cleansing, use of antimicrobial suture technology at wound closure and embracing innovative antimicrobial surgical glove technology reducing the risk of bacterial passage into the surgical wound.

## Disclosure of interest

None declared.

